# Radiomics Nomogram: Prediction of 2-Year Disease-Free Survival in Young Age Breast Cancer

**DOI:** 10.3390/cancers14184461

**Published:** 2022-09-14

**Authors:** Jeongmin Lee, Sung Hun Kim, Yelin Kim, Jaewoo Park, Ga Eun Park, Bong Joo Kang

**Affiliations:** 1 Department of Radiology, College of Medicine, Seoul Saint Mary’s Hospital, The Catholic University of Korea, Seoul 06591, Korea; 2 Department of Statistics and Data Science, Yonsei University, 50 Yonsei-ro, Seodaemun-gu, Seoul 06591, Korea; 3 Department of Applied Statistics, Yonsei University, 50 Yonsei-ro, Seodaemun-gu, Seoul 06591, Korea

**Keywords:** young age breast cancer, disease-free survival, imaging biomarker, breast MRI, radiomics nomogram

## Abstract

**Simple Summary:**

Breast cancer in young women under 40 years of age shows a poor prognosis, and its treatment is difficult due to premenopausal status and fertility preservation. The early prediction of prognosis of young age breast cancer would be helpful for planning treatment and postoperative surveillance. In this study, the radiomics-based nomogram for the prediction of recurrence shows good predictive ability, especially 2-year disease-free survival after surgery. Several radiomics features presumed to be unique imaging features of young age breast cancer were also observed: tumor homogeneity and tumor sphericity. As these radiomics features are quantitative parameters extracted through the texture analysis of breast MRI, they may reflect the information of tumors more objectively, such as the tumor microenvironment. Furthermore, these results will be the basis for identifying the unique biology of young age breast cancer through multi-omics studies such as radio-genomics.

**Abstract:**

This study aimed to predict early breast cancer recurrence in women under 40 years of age using radiomics signature and clinicopathological information. We retrospectively investigated 155 patients under 40 years of age with invasive breast cancer who underwent MRI and surgery. Through stratified random sampling, 111 patients were assigned as the training set, and 44 were assigned as the validation set. Recurrence-associated factors were investigated based on recurrence within 5 years during the total follow-up period. A Rad-score was generated through texture analysis (3D slicer, ver. 4.8.0) of breast MRI using the least absolute shrinkage and selection operator Cox regression model. The Rad-score showed a significant association with disease-free survival (DFS) in the training set (*p* = 0.003) and validation set (*p* = 0.020) in the Kaplan–Meier analysis. The nomogram was generated through Cox proportional hazards models, and its predictive ability was validated. The nomogram included the Rad-score and estrogen receptor negativity as predictive factors and showed fair DFS predictive ability in both the training and validation sets (C-index 0.63, 95% CI 0.45–0.79). In conclusion, the Rad-score can predict the disease recurrence of invasive breast cancer in women under 40 years of age, and the Rad-score-based nomogram showed reasonably high DFS predictive ability, especially within 2 years of surgery.

## 1. Introduction

For decades, the development of treatment and screening systems for breast cancer has contributed to improving breast cancer mortality rates [1,2,3]. Despite these improvements, the incidence of breast cancer and cancer-associated mortality in Asia has rapidly increased, and the incidence and mortality rates of breast cancer were the highest in Asia among five continents in 2020 [4,5]. Moreover, breast cancer in Asia affects a distinct population. The peak age of Asian women with breast cancer is mid-40s, much younger than that of women from Western countries (in the 70s), and the proportion of young women is also much higher in Asia [5,6]. In addition, aggressive tumor biology, including higher proportions of the triple-negative subtype in young age breast cancer (YABC), has been confirmed by several studies, and a young age is now regarded as a poor prognostic factor for breast cancer [7,8,9]. In the prognosis of young age breast cancer, the association with family history or genetic mutations such as BRCA1/BRCA2 is always mentioned in YABC. However, most YABC patients have no first-degree family history of breast cancer (89%), only 10% of YABC patients are BRCA carriers and most YABC cases occur sporadically [10,11]. Consequently, there is controversy regarding whether YABC should be classified as a distinct type among various subtypes of breast cancer.

Standard screening programs and treatment guidelines for breast cancer are currently based on the data of breast cancer patients of average age, especially in the Western hemisphere. In addition, regardless of the continent, most of the previous studies evaluated breast cancer in average-age women. With the recent increase in the incidence of breast cancer in Asia, several clinical studies have investigated the characteristics of YABC. However, most studies have focused on the treatment methods or molecular characteristics of Asian YABC [12,13], and only a few studies on the imaging characteristics of YABC have been published. A previous study investigated the imaging features of YABC, focusing on the correlation of pathologic factors: a high recurrence rate within two years of breast cancer surgery and recurrence-associated MRI parameters were revealed through texture analysis [14]. Radiomics, which is defined as the extraction and analysis of high-dimensional quantitative data from medical imaging, is an emerging translational field of medicine. The traditional imaging evaluation of tumors through mammography, ultrasound or MRI is qualitative, and it may vary depending on the experience of the radiologists. On the other hand, the radiologists can assess more objective imaging features through radiomics-based tumor analysis [15,16], and radiomics-based analysis is applied to various imaging methods, including mammography, ultrasound and MRI [17,18,19,20].

Considering this background, we aimed to utilize the recurrence-associated imaging features to create the radiomics signature of YABC and establish a nomogram for the prediction of disease-free survival (DFS) of YABC using radiomics signature and variable clinical factors.

## 2. Materials and Methods

### 2.1. Patients

This retrospective study was approved by the Institutional Review Board of our institution. The requirement for informed consent was waived by the ethics committee due to the retrospective design. All assessments were carried out according to the Declaration of Helsinki of 1975, revised in 2013.

From January 2011 to February 2019, among 4451 patients who underwent breast cancer surgery in our institution, 320 female patients under 40 years old who were diagnosed with invasive breast cancer were consecutively included. Because pretreatment breast MRI was used to create the radiomics signature, patients without pretreatment MRI (*n* = 21) or patients with incomplete MR data (*n* = 86) were excluded. To investigate the DFS rates two years after surgery, patients with less than two years of follow-up (*n* = 58) were excluded. Patients who had not been diagnosed with recurrent breast cancer by the end of five years were censored.

Disease recurrence was defined as newly diagnosed breast cancer in the ipsilateral or contralateral breast or the axillary or distant metastasis. The diagnosis of disease recurrence was based on pathological confirmation through biopsy. If biopsy was impossible, recurrence was diagnosed using imaging modalities such as positron emission tomography and computed tomography.

### 2.2. MRI Protocol

All enrolled patients underwent dynamic contrast-enhanced MRI, including diffusion-weighted images with 3-Teslar MRI vendors. MRI was performed in the prone position using a dedicated bilateral breast surface coil. Imaging with 3-Teslar MRI systems (Verio, Siemens Healthcare, Erlangen, Germany; Ingenia, Philips Medical Systems, Best, The Netherlands) was performed. The detail of each sequence is described in Appendix A.1.

### 2.3. Texture Analysis for Radiomics Feature Extraction

Two expert breast radiologists with 8 and 24 years of experience in breast MRI retrospectively reviewed the pretreatment breast MRI using a picture archiving and communication system (PACS) with a workstation monitor. The target lesion was defined as the largest enhancing lesion regardless of mass or non-mass enhancement. One radiologist segmented the target lesion three-dimensionally with a semiautomatic tool using an open-source software package (3D slicer, ver. 4.10.2; available at: https://slicer.org/, accessed on 1 June 2020) in the early phase of contrast-enhanced T1-weighted subtraction imaging and ADC mapping. If the target lesion was not clearly delineated on the ADC map, the segmented volume of interest on early-phase contrast-enhanced T1 subtraction images was applied to the ADC map with modification. After the segmentation of the target lesion, radiomics features were extracted using open-source PyRadiomics software. A total of 107 features were extracted from early-phase contrast-enhanced T1 subtraction images and ADC maps.

### 2.4. Clinicopathologic Information and Conventional MRI Analysis

Clinicopathologic information including age, treatment methods, stage and follow-up period after surgery were reviewed from medical records. Pathologic characteristics of tumors were collected from pathologic reports after surgery (Appendix A).

Morphologic data of the tumor, including shape, margin, internal enhancement pattern and enhancement kinetics, were obtained from the expert consensus of the conventional MRI analysis of PACS and reviewed according to the fifth edition of the Breast Imaging Reporting and Data System MRI lexicon. In addition, previously established poor prognostic factors, peritumoral edema and ipsilateral vascularity around the tumor [21,22], were reviewed on T2-weighted images and maximum intensity projection images, respectively.

### 2.5. Statistical Analysis

As in the standard statistical inference, two-tailed *p* values < 0.05 were considered significant. All statistical analyses were performed using R software version 4.0.2 (Ihaka and Gentleman, 1996). We fitted the least absolute shrinkage and selection operator (LASSO) Cox regression model using the “glmnet” package, and the “rms” and “hdnom” packages were used for the Kaplan–Meier curve, nomogram and calibration plot.

#### Creation and Validation of Rad-Score

We randomly divided patients into a training set (*n* = 111) and a validation set (*n* = 44) to create a radiomics signature (Rad-score). To compare patients’ characteristics between the two cohorts, we conducted a Wilcoxon rank-sum test for continuous variables and Fisher’s exact test for categorical variables. We first used the univariate Cox proportional hazards model to screen for insignificant variables. We then used the LASSO regularization method [23] to perform radiomics feature selection from the training set. The Rad-score of each patient was calculated using a combination of selected features and their estimated coefficients. We analyzed the association between the Rad-score and DFS from the training set and then assessed it in the validation set. The patients were classified as high- or low-risk based on the Rad-score. We used Youden’s index [24] to set the cut-off value of the Rad-score in the receiver-operating characteristic curve analysis. The DFS of high- and low-risk patients was analyzed using Kaplan–Meier survival analysis, and the log-rank test was applied to evaluate the difference in DFS between the two groups.

A radiomics-based prediction model for recurrence was constructed using the Rad-score and clinicopathologic and morphologic information. We used the univariate Cox proportional hazards model to exclude the insignificant variables. We then fitted the multivariate Cox proportional hazards model based on all significant variables from the univariate analysis. To avoid multicollinearity, we conducted stepwise variable selection based on the Akaike information criterion. We compared the prediction power among the models with several combinations of significant variables in the validation set with the C-index and calibrated them.

## 3. Results

### 3.1. Patient Characteristics

**Figure 1:**
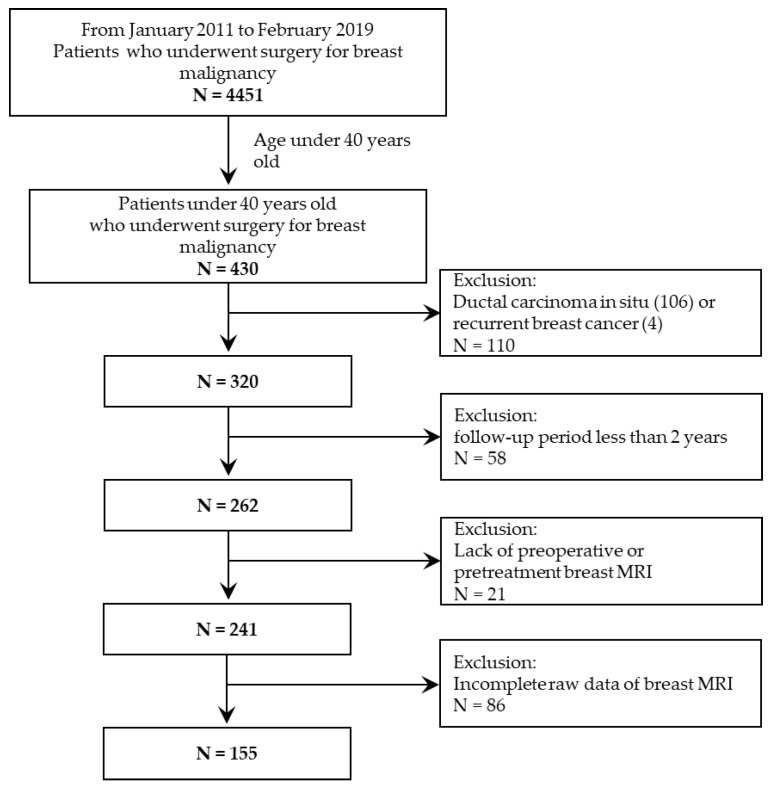
Patient inclusion and exclusion criteria.

In total, 155 patients were enrolled in the study (Figure 1). The mean age was 35 years (SD ± 4.8), and the mean follow-up period was 55 months (SD ± 26.55). Of the 155 patients, 138 were diagnosed with invasive ductal carcinoma, 4 were diagnosed with invasive lobular carcinoma and 13 were diagnosed with other histologies, including mucinous carcinoma (8 cases), adenoid cystic carcinoma (2 cases), invasive micropapillary carcinoma (1 case), metaplastic carcinoma (1 case), and mixed mucinous and invasive micropapillary carcinoma (1 case). There were 50 patients with luminal A subtype (32.2%), 61 patients with luminal B subtype (39.4%), 10 patients with HER2 positive subtype (6.5%) and 34 patients with triple-negative subtype (21.9%). Furthermore, 52 patients underwent neoadjuvant chemotherapy after diagnosis, while the remaining 103 patients did not.

During the mean follow-up period of 55 months (6–118 months), 42 patients were diagnosed as experiencing recurrence. Among the 42 recurrent patients, 3 were diagnosed as experiencing recurrence after five years (60 months) of surgery and were censored. Among the 39 recurrent patients, 20 (13 in the training set and 7 in the validation set) experienced disease recurrence within 24 months, with a median recurrence interval of 13 months (range, 3–23 months). Of the 20 cases of recurrence, 13 were manifested as local recurrence of the ipsilateral breast, 6 were manifested as distant metastases to the lung, brain and contralateral supraclavicular lymph nodes, 1 was manifested as contralateral breast recurrence, 1 was manifested as both ipsilateral breast recurrence and distant metastasis, and 1 was manifested as both contralateral breast recurrence and distant metastasis. The patients’ characteristics in each cohort are listed in Table 1. Except for delayed enhancement kinetics, there was no difference between the training and validation sets.

### 3.2. Creation of Rad-Score & Assessment of Disease-Free Survival

The 214 total texture features (107 from each cohort), including shape (14 features), first-order (18 features), gray-level co-occurrence matrix (24 features), gray-level run-length matrix (16 features), gray-level size zone matrix (16 features), gray-level dependence matrix (14 features) and neighboring gray-tone difference matrix (5 features), were extracted. These features were standardized and used to generate the radiomics-based scoring equation (Rad-score). Based on the fitted LASSO Cox regression model from the training set, the Rad-score for predicting disease recurrence was created as follows:

$$\begin{array}{cc} \mathrm{Rad}-\mathrm{score}= & \left(-0.08303501\times \mathrm{surface}\mathrm{volume}\mathrm{ratio}\_T1\right)\\ & +(0.19940815\times \mathrm{Large}\mathrm{Area}\mathrm{Low}\mathrm{Gray}\mathrm{Level}\mathrm{Emphasis}\_T1)\\ & +(0.1041660\times \mathrm{correlation}\_ADC)\\ & +(0.06086060\times \mathrm{Cluster}\mathrm{Prominence}\_ADC)\\ & +(0.04198567\times \mathrm{Cluster}\mathrm{Tendency}\_ADC)\\ & +\left(0.12402528\times High\;Gray\;Level\;Zone\;Emphasis\_ADC\right). \end{array}$$

Using the equation above, the Rad-score of each patient was calculated, and the patients in the training set were classified as high- or low-risk for disease recurrence. The optimal cut-off value for separating the high- and low-risk patients was determined to be −0.016 according to Youden’s index. The characteristics of the patients according to risk are presented in Table 2. In the training set, Rad-score, mastectomy ratio, T stage, N stage, overall stage, mean tumor size, ratio of non-mass enhancement or combined pattern, ipsilateral vascularity and the presence of peritumoral edema were all positively correlated with high risk.

**Figure 2:**
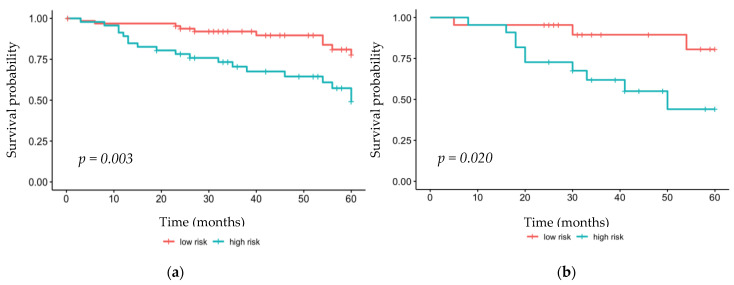
Kaplan–Meier analysis of the (**a**) training set and (**b**) validation set.

Using Kaplan–Meier survival analysis, the DFS by Rad-score was analyzed. The high-risk group in the training set showed significantly lower DFS values within 2 years of surgery (*p* = 0.003). Similarly, the high-risk group showed significantly lower survival rates in the validation set (*p* = 0.020) (Figure 2).

### 3.3. Rad-Score-Based Recurrence Prediction Model: Radiomics Nomogram

**Figure 3:**
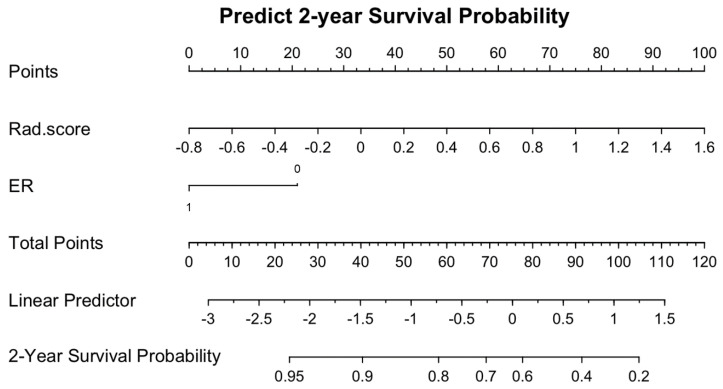
Radiomics-based nomogram for the prediction of two-year disease recurrence in young age breast cancer.

Univariate analysis showed that a higher Rad-score (*p* < 0.001) and ER-negativity (*p* = 0.044) were associated with recurrence. Multivariate analysis confirmed the independent association of Rad-score (hazard ratio 5.87, 95% CI 2.87–11.99, *p* < 0.001) and ER-negativity (hazard ratio 0.41, 0.19–0.86, *p* = 0.019) with disease recurrence (Table 3). With Rad-score and ER-negativity, the Rad-score-based nomogram for the prediction of disease recurrence within 2 years of surgery was created (Figure 3).

**Figure 4:**
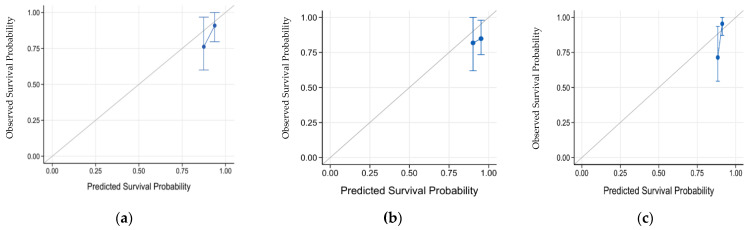
Calibration curves of three models in the validation set. (**a**) Calibration curve of the radiomics nomogram. (**b**) Calibration curve of the ER-negativity-only model. (**c**) Calibration curve of the Rad-score-only model. (ER: estrogen receptor.)

**Figure 5:**
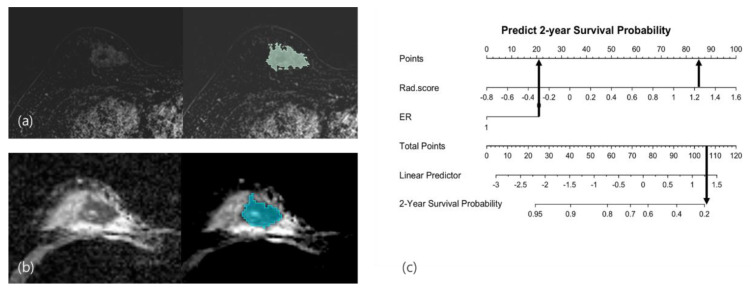
Recurrence case: A 34-year-old woman with grade 3 invasive breast cancer of the triple-negative subtype had an irregularly shaped mass with internal heterogeneous enhancement in the right breast. After segmentation of the mass in (**a**) the contrast-enhanced subtraction T1-weighted image and (**b**) the ADC map, texture analysis was performed, and the Rad-score was calculated as 1.253. (**c**) Applying this score and ER-negativity in the radiomics nomogram, the total points were calculated to be 115, which showed a less-than-20% probability of recurrence survival. The patient underwent nipple-sparing mastectomy with the reconstruction of the right breast, and skin metastasis was confirmed in the right reconstructed breast three months after surgery. (ER: estrogen receptor; ADC: apparent diffusion coefficient.)

**Figure 6:**
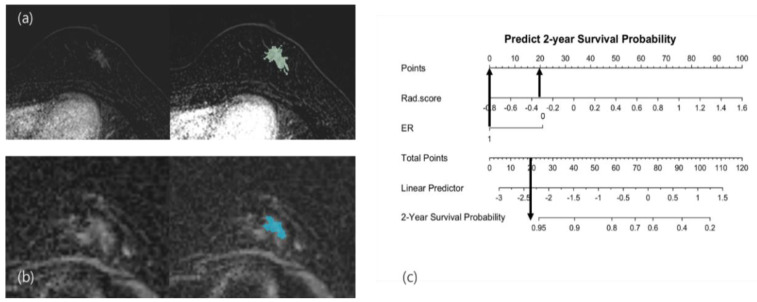
Nonrecurrence case: A 37-year-old woman with grade 2 invasive breast cancer of the hormone receptor-positive subtype. After segmentation of the mass in (**a**) the contrast-enhanced subtraction T1-weighted images and (**b**) the ADC map, the Rad-score based on texture analysis was calculated as −0.345. (**c**) Applying this score and ER-positivity in the radiomics nomogram, the total points were calculated as 20, which showed a probability of recurrence survival of more than 95%. The patient underwent breast-conserving surgery with adjuvant chemotherapy and hormone therapy. The patient remained stable, without recurrence. (ER: estrogen receptor; ADC: apparent diffusion coefficient.)

**Figure A1:**
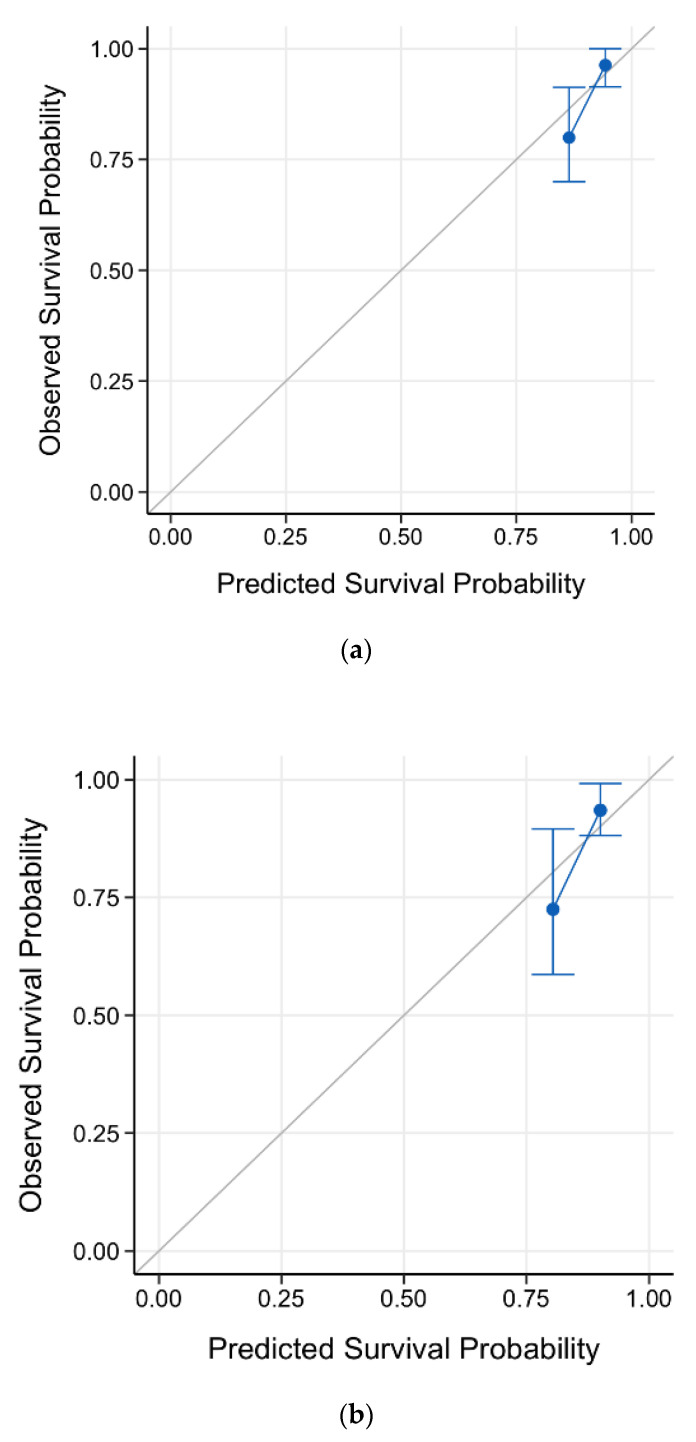

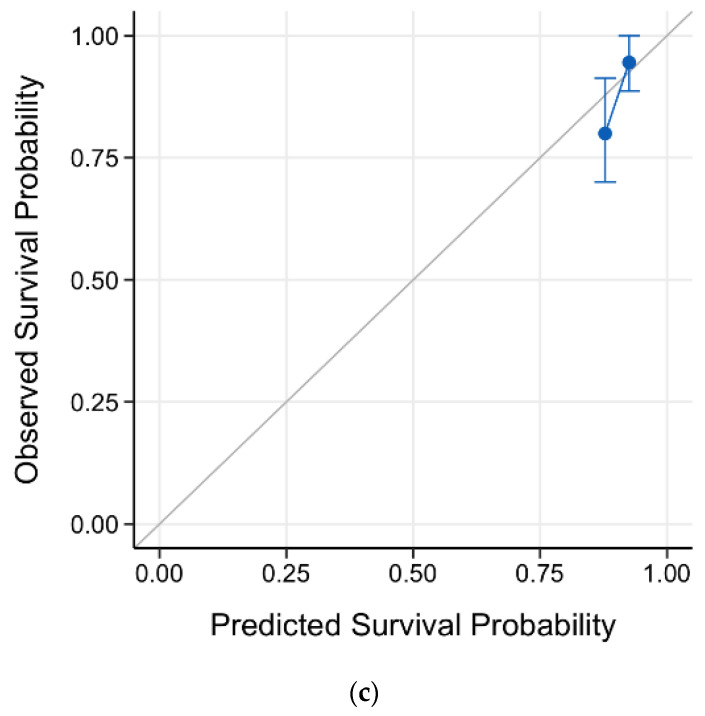
(**a**) Calibration curve of the radiomics nomogram in the training set; (**b**) Calibration curve of the ER-negativity-only model in the training set; (**c**) Calibration curve of the Rad-score-only model in the training set.

We compared the prediction power among three models in the validation set: the radiomics nomogram, the ER-negativity only model and the Rad-score only model. The C-index of the radiomics nomogram was 0.63 (95% CI 0.45–0.80), displaying fair predictive ability for disease recurrence, whereas the C-index of the ER-negativity-only model was 0.51 (95% CI 0.39–0.66). In addition, the Rad-score-only model showed a value of 0.71 (95% CI 0.51–0.86). The calibration curves of these models in the validation set are shown in Figure 4. Additionally, the calibration curves of these models in the training set are shown in Figure A1, Appendix B. Representative recurrent and non-recurrent cases are shown in Figure 5 and Figure 6.

## 4. Discussion

In this study, we developed a radiomics-based nomogram for predicting early disease recurrence in YABC. Rad-score and ER negativity were associated with early cancer recurrence within two years of surgery. Among the three prediction models, those employing the Rad-score demonstrated higher predictive ability for recurrence than the model that only included ER status. Moreover, the Rad-score-only model showed a higher predictive performance than the Rad-score and ER negativity model. Note that the relatively small sample size may lead to larger variability in predictive ability; the sparse model is often preferred in this case. However, as we pointed out, the sample size used in this study can still provide a small margin of error close to the threshold value suggested in [25].

The Rad-score was generated using the equation created from six features of the tumor texture analysis. Of these six features, a few were consistent with a previous study on YABC. Previously, a low surface-area-to-volume ratio, indicating tumor sphericity, and texture parameters indicating tumor homogeneity exhibited an association with cancer recurrence [14]. In this study, a low surface-area-to-volume ratio and a high cluster tendency were also correlated with a high Rad-score. Moreover, most malignant masses exhibited a high surface-area-to-volume ratio because of irregular shapes with non-circumscribed margins. In contrast, a low surface-area-to-volume ratio indicated a more spherical shape. This result is not only consistent with previous studies but is also consistent with the fact that the triple-negative subtype tends to be more spherical [26,27], and there was a high proportion of the triple-negative subtype in the recurrence group in the current study.

Generally, tumor heterogeneity from texture analysis is a poor prognostic factor, as it represents aggressive tumor biology [28,29]. However, several previous studies suggested that lower entropy or higher tumor uniformity in contrast-enhanced T1 subtraction images, as well as tumor heterogeneity in T2-weighted images, are associated with poor breast cancer outcomes [29,30]. In these previous studies, it was hypothesized that the vascular permeability of tumors leads to increased parenchymal enhancement, resulting in less heterogeneity in texture analysis. However, the results of the present and previous studies consistently showed an association between the tumor homogeneity of ADC maps and lower DFS. In this study, the cluster tendency from the ADC map showed a positive correlation with disease recurrence, and in a previous study, the inverse difference moment from the ADC map was associated with disease recurrence. Usually, ADC maps represent tumor cellularity, and low ADC values are associated with high-grade tumors [31] or high tumor proliferation [32]. However, because the association between tumor cellularity and the texture parameters of ADC maps has not yet been evaluated, tumor homogeneity as a recurrence-associated factor is not yet confirmed as a unique factor of YABC. Technically, variations between MRI vendors can affect the tumor homogeneity from texture analysis. Therefore, further studies are warranted to verify tumor homogeneity from ADC maps as a recurrence-associated factor in YABC.

Of the 20 cases of disease recurrence, 10 showed ER-negativity, 2 of which were HER2 positive and 8 were triple-negative. Because we investigated DFS within two years of surgery, the high proportion of the triple-negative subtype is expected due to the aggressive nature of the triple-negative subtype [33,34]. In our study, the overall rate of the triple-negative subtype was higher (22%, 34/155) than that of the general population of breast cancer, which is consistent with the idea that YABC has a higher rate of the triple-negative subtype [35]. Thus, ER-negativity as a recurrence-associated factor cannot necessarily be considered unique to YABC. Therefore, further studies are needed to compare clinical, imaging or genetic features of ER-negative breast cancer between young- and average-age breast cancer patients.

This study has the limitation of the inevitable selection bias of YABC cases because it was a retrospective study conducted at a single institution. Second, a relatively small sample size was used to make and validate the nomogram. Moreover, the Rad-score-only model showed a higher predictive performance than the radiomics nomogram model, which has both the Rad-score and ER negativity as predictors. Note that the relatively small sample size may lead to a larger variability in prediction; in this case, the model with a smaller number of predictors is often preferred in the test dataset. Therefore, we calculate a margin of error and compare it with the guidelines provided in [25]. For a given sample of 155 with a 12.9% recurrence rate, the margin of error is 0.053, which is close to 0.05, the suggested threshold value in [25]. This implies that the sample size used in this study can lead to robust prediction models, though collecting larger samples could provide a smaller variability in prediction. We provide details about calculating the margin of error in Appendix C. In addition, considering the proportion of young age patients among all breast cancer patients, the number of patients enrolled in our study is not that small. Third, though we validated the nomogram with a separate cohort, we did not perform external validation with data from more independent resources, such as prospective patient groups or patient groups of other institutions. For this reason, we are planning to validate this nomogram with a prospective group in our institution. Moreover, the overall survival of YABC should be investigated with a follow-up period of more than 10 years. Finally, information regarding family history or the presence of BRCA mutations is lacking in our study group. However, only 10% of YABC patients have an association with a first-degree family history of breast cancer or BRCA mutations [10,11], and a family history of breast cancer or the presence of BRCA mutations cannot affect the mortality of breast cancer patients [36,37].

## 5. Conclusions

In conclusion, our nomogram based on the radiomics signature and clinicopathologic information showed reasonably high predictive ability of disease-free survival, especially within 2 years of surgery. Future prospective studies should be conducted to validate the predictability of the radiomics nomogram for YABC. Furthermore, it is crucial to determine the relationship between tumor biology including genetic mutation and the imaging phenotype of YABC through multi-omics studies.

## Figures and Tables

**Table 1:** Characteristics of patients in the training set and validation set.

Characteristics	Training Set (*n* = 111)	Validation Set (*n* = 44)	*p* Value
Age	34.94 ± 5.25	35.73 ± 3.48	0.511
Rad-score	−0.01 ± 0.36	0.04 ± 0.45	0.358
Operation			0.103
Breast-conserving surgery	70 (63.06%)	21 (47.73%)	
Mastectomy	41(36.94%)	23 (52.27%)	
Adjuvant radiation therapy			0.237
Yes	95 (85.59%)	34 (77.27%)	
No	16 (14.41%)	10 (22.73%)	
Adjuvant chemotherapy			0.537
Yes	86 (77.48%)	32 (72.73%)	
No	25 (22.52%)	12 (27.27%)	
Adjuvant endocrine therapy			0.549
Yes	79 (71.17%)	34 (77.27%)	
No	32 (28.83%)	10 (22.73%)	
Adjuvant target therapy			0.624
Yes	16 (14.41%)	8 (18.18%)	
No	95 (85.59%)	36 (81.82%)	
T stage			1
1	35 (31.53%)	14 (31.82%)	
2	55 (49.55%)	21 (47.73%)	
3	19 (17.12%)	8 (18.18%)	
4	2 (1.80%)	1 (2.27%)	
N stage			0.780
0	49 (44.14%)	23 (52.27%)	
1	41 (36.94%)	13 (29.55%)	
2	12 (10.81%)	4 (9.09%)	
3	9 (8.11%)	4 (9.09%)	
M stage			1
0	110 (99.10%)	44 (100%)	
1	1 (0.90%)	0 (0%)	
Overall stage			0.921
I	24 (21.62%)	10 (22.73%)	
II	59 (53.15%)	22 (50%)	
III	27 (24.32%)	12 (27.27%)	
IV	1 (0.90%)	0 (0%)	
Histologic type			0.537
Invasive breast cancer	97 (87.39%)	41 (93.18%)	
Invasive lobular carcinoma	4 (3.60%)	0 (0%)	
Others	10 (9.01%)	3 (6.82%)	
Histologic grade			0.843
1	20 (18.02%)	8 (18.18%)	
2	53 (47.75%)	19 (43.18%)	
3	38 (34.23%)	17 (38.64%)	
Lymphovascular invasion			0.858
Present (yes)	48 (43.24%)	20 (45.45%)	
Absent (no)	63 (56.76%)	24 (54.55%)	
Estrogen receptor			0.693
Positive	78 (70.27%)	33 (75%)	
Negative	33 (29.73%)	11 (25%)	
Progesterone receptor			0.717
Positive	66 (59.46%)	28 (63.64%)	
Negative	45 (40.54%)	16 (36.36%)	
HER2			0.461
Positive	15 (13.51%)	8 (18.18%)	
Negative	96 (86.49%)	36 (81.82%)	
Ki67 index			0.894
Mean (± SD)	36.06 ± 27.46	36.23 ± 27.24	
Tumor size			0.250
Mean (± SD)	3.59 ± 2.27	4.05 ± 2.48	
Tumor laterality			0.481
Right	62 (55.86%)	22 (50%)	
Left	48 (43.24%)	22 (50%)	
Lesion type			0.866
Mass	80 (72.07%)	33 (75%)	
Non-mass enhancement (NME)	9 (8.11%)	2 (4.55%)	
Mass with NME	22 (19.82%)	9 (20.45%)	
Peritumoral edema on T2WI			0.722
Present (yes)	53 (47.75%)	19 (43.18%)	
Absent (no)	58 (52.25%)	25 (56.82%)	
Ipsilateral vascularity			0.102
Mean (± SD)	3.29 ± 2.7	3.82 ± 2.6	
Multifocality			1
Yes	54 (48.65%)	22 (50%)	
No	57 (51.35%)	22 (50%)	
Early enhancement pattern			0.482
Rapid	105 (94.59%)	44 (100%)	
Medium	2 (1.80%)	0 (0%)	
Slow	4 (3.60%)	0 (0%)	
Delayed enhancement pattern			0.005
Washout	80 (72.07%)	32 (72.73%)	
Plateau	16 (14.41%)	12 (27.27%)	
Persistent	15 (13.51%)	0 (0%)	
Fibroglandular tissue			0.617
Fatty	0 (0%)	0 (0%)	
Scattered	5 (4.50%)	2 (4.55%)	
Heterogenous	75 (67.57%)	33 (75%)	
Extreme	31 (27.93%)	9 (20.45%)	
Background parenchymal enhancement			1
Minimal	51 (45.95%)	20 (45.45%)	
Mild	21 (18.92%)	8 (18.18%)	
Moderate	26 (23.42%)	11 (25%)	
Marked	13 (11.71%)	5 (11.36%)	

**Table 2:** Characteristics of patients in the low-risk and high-risk groups in the training set.

Characteristics	Low-Risk Group (*n* = 65)	High-Risk Group (*n* = 46)	*p* Value
Age	35.05 ± 6.09	34.78 ± 3.82	0.141
Rad-score	−0.22 ± 0.14	0.27 ± 0.38	<0.001
Operation			<0.001
Breast conserving surgery	51 (78.46%)	19 (41.30%)	
Mastectomy	14 (21.54%)	27 (58.70%)	
Adjuvant radiation therapy			0.585
Yes	57 (87.69%)	38 (81.61%)	
No	8 (12.31%)	8 (17.39%)	
Adjuvant chemotherapy			0.494
Yes	52 (80%)	34 (73.91%)	
No	13 (20%)	12 (26.09%)	
Adjuvant endocrine therapy			0.833
Yes	47 (72.31%)	32 (69.57%)	
No	18 (27.69%)	14 (30.43%)	
Adjuvant target therapy			0.273
Yes	7 (10.77%)	9 (19.57%)	
No	58 (89.23%)	37 (80.43%)	
T stage			<0.001
1	32 (49.23%)	3 (6.52%)	
2	31 (47.69%)	24 (52.17%)	
3	2 (3.08%)	17 (36.96%)	
4	0 (0%)	2 (4.35%)	
N stage			0.003
0	33 (50.77%)	16 (34.78%)	
1	27 (41.54%)	14 (30.43%)	
2	4 (6.15%)	8 (17.39%)	
3	1 (1.54%)	8 (17.39%)	
M stage			1
0	64 (98.46%)	46 (100%)	
1	1 (1.54%)	0 (0%)	
Overall stage			<0.001
I	22 (33.85%)	2 (4.35%)	
II	36 (55.38%)	23 (50%)	
III	6 (9.23%)	21 (45.65%)	
IV	1 (1.54%)	0 (0%)	
Histologic type			0.740
Invasive breast cancer	57 (87.69%)	40 (86.96%)	
Invasive lobular carcinoma	3 (4.62%)	1 (2.17%)	
Others	5 (7.69%)	5 (10.87%)	
Histologic grade			0.844
1	13 (20%)	7 (15.22%)	
2	30 (46.15%)	23 (50%)	
3	22 (33.85%)	16 (34.78%)	
Lymphovascular invasion			0.846
Present (yes)	29 (44.62%)	19 (41.30%)	
Absent (no)	36 (55.38%)	27 (58.70%)	
Estrogen receptor			0.207
Positive	49 (75.38%)	29 (63.04%)	
Negative	16 (24.62%)	17 (36.96%)	
Progesterone receptor			0.240
Positive	42 (64.62%)	24 (52.17%)	
Negative	23 (35.38%)	22 (47.83%)	
HER2			0.159
Positive	6 (9.23%)	9 (19.57%)	
Negative	59 (90.77%)	37 (80.43%)	
Ki67 index			0.636
Mean (±SD)	34.34 ± 26.38	38.49 ± 29.04	
Tumor size			<0.001
Mean (±SD)	2.74 ± 1.84	4.8 ± 2.28	
Tumor laterality			0.172
Right	40 (61.54%)	22 (47.83%)	
Left	24 (36.92%)	24 (52.17%)	
Lesion type			0.026
Mass	53 (81.54%)	27 (58.70%)	
Non-mass enhancement (NME)	4 (6.15%)	5 (10.87%)	
Mass with NME	8 (12.31%)	14 (30.43%)	
Peritumoral edema on T2WI			<0.001
Present (yes)	22 (33.85%)	31 (67.39%)	
Absent (no)	43 (66.15%)	15 (32.61%)	
Ipsilateral vascularity			<0.001
Mean (±SD)	2.42 ± 1.98	4.5 ± 3.09	
Multifocality			0.086
Yes	27 (41.54%)	27 (58.70%)	
No	38 (58.46%)	19 (41.30%)	
Early enhancement pattern			0.824
Rapid	61 (93.85%)	44 (95.65%)	
Medium	1 (1.54%)	1 (2.17%)	
Slow	3 (4.62%)	1 (2.17%)	
Delayed enhancement pattern			0.785
Washout	48 (73.85%)	32 (69.57%)	
Plateau	8 (12.31%)	8 (17.39%)	
Persistent	9 (13.85%)	6 (13.04%)	
Fibroglandular tissue			0.239
Fatty	0 (0%)	0 (0%)	
Scattered	1 (1.54%)	4 (8.70%)	
Heterogenous	46 (70.77%)	29 (63.04%)	
Extreme	18 (27.69%)	13 (28.26%)	
Background parenchymal enhancement			0.466
Minimal	32 (49.23%)	19 (41.30%)	
Mild	12 (18.46%)	9 (19.57%)	
Moderate	12 (18.46%)	14 (30.43%)	
Marked	9 (13.85%)	4 (8.70%)	

**Table 3:** Univariate and multivariate analysis of DFS in the training set.

	Univariate Analysis		Multivariate Analysis	
Characteristics	Hazard Ratio (95% CI)	*p* Value	Hazard Ratio (95% CI)	*p* Value
Age	1.00 (0.93–1.07)	0.985		
Rad-score	5.15 (2.60–10.20)	<0.001	5.87 (2.87–11.99)	<0.001
Operation				
Breast-conserving surgery	Ref			
Mastectomy	1.02 (0.48–2.16)	0.96		
Adjuvant radiation therapy				
Yes	0.52 (0.21–1.28)	0.154		
No	Ref			
Adjuvant chemotherapy				
Yes	0.89 (0.332–2.386)	0.817		
No	Ref			
Adjuvant endocrine therapy				
Yes	0.48 (0.23–0.99)	0.05		
No	Ref			
Adjuvant target therapy				
Yes	1.38 (0.52–3.62)	0.512		
No	Ref			
T stage				
1	Ref			
2	2.52 (0.91–7.0)	0.075		
3	3.09 (1.01–9.46)	0.048		
4	7.94 (0.90–70.18)	0.063		
N stage				
0	Ref			
1	0.53 (0.64–3.63)	0.337		
2	1.36 (0.37–5.0)	0.647		
3	5.13 (1.71–15.39)	0.003		
M stage				
0	Ref			
1	0 (0-∞)	0.997		
Stage				
I	Ref			
II	1.79 (0.59–5.40)	0.301		
III	2.66 (0.83–8.47)	0.099		
IV	0 (0-∞)	0.997		
Histologic type				
Invasive breast cancer	Ref			
Invasive lobular carcinoma	0 (0-∞)	0.997		
Others	1.23 (0.37–4.06)	0.737		
Histologic grade				
1	Ref			
2	1.6 (0.45–5.62)	0.463		
3	2.39 (0.68–8.34)	0.174		
Lymphovascular invasion				
Present (yes)	0.75 (0.36–1.56)	0.455		
Absent (no)	Ref			
Estrogen receptor				
Positive	0.47 (0.23–0.98)	0.044	0.41 (0.19–0.86)	0.019
Negative	Ref		Ref	
Progesterone receptor				
Positive	0.514 (0.247–1.07)	0.075		
Negative	Ref			
HER2				
Positive	2.043 (0.829–5.035)	0.12		
Negative	Ref			
Ki67 index	1.011 (0.998–1.024)	0.094		
Tumor size	1.119 (0.972–1.287)	0.118		
Tumor laterality				
Right	Ref			
Left	1.199 (0.579–2.485)	0.625		
Lesion type				
Mass	Ref			
Non-mass enhancement (NME)	0.605 (0.141–2.595)	0.499		
Mass with NME	0.935 (0.377–2.318)	0.885		
Peritumoral edema on T2WI				
Present (yes)	1.873 (0.884–3.967)	0.101		
Absent (no)	Ref			
Ipsilateral vascularity	1.124 (0.995–1.269)	0.06		
Multifocality				
Yes	0.942 (0.453–1.957)	0.873		
No	Ref			
Early enhancement pattern				
Rapid	Ref			
Medium	1.999 (0.27–14.778)	0.497		
Slow	1.311 (0.18–9.66)	0.79		
Delayed enhancement pattern				
Washout	Ref			
Plateau	1.158 (0.43–3.11)	0.77		
Persistent	1.469 (0.55–3.94)	0.445		
Fibroglandular tissue				
Fatty	NA			
Scattered	Ref			
Heterogenous	0.625 (0.14–2.71)	0.529		
Extreme	0.815 (0.18–3.73)	0.792		
Background parenchymal enhancement				
Minimal	Ref			
Mild	1.54 (0.62–3.87)	0.356		
Moderate	0.95 (0.38–2.38)	0.909		
Marked	0.53 (0.12–2.37)	0.41		

## Data Availability

All data generated and analyzed during this study are included in this published article. Raw data supporting the findings of this study are available from the corresponding author on request.

## Appendix A. Material & Methods

### Appendix A.1. Protocols of MRI

#### Appendix A.1.1. Verio, Siemens Healthcare

(1) Axial, turbo spin-echo T2-weighted-imaging sequence with a TR/TE of 4530/93, a flip angle of 80°, 34 slices, a 320 mm field of view, a matrix size of 576 × 403, 1 excitation, a 4 mm slice thickness and an acquisition time of 2 min 28 s; (2) axial diffusion-weighted imaging with a single-shot echoplanar image or a readout-segmented echoplanar image (b values 0 and 750 s/mm^2^, TR/TE 9800/87 ms and 5600/55 ms, respectively; field of view, 340 × 117 mm^2^ and 360 × 180 mm^2^, respectively; matrix size, 192 × 82; slice thickness 4 mm; acquisition time 2 min 47 s and 2 min 31 s, respectively; 5 readout segments for the readout-segmented echoplanar image). Apparent diffusion coefficient (ADC) maps were calculated automatically using MRI software; (3) pre- and post-contrast axial T1-weighted flash 3D volumetric interpolated brain examination sequences with a TR/TE of 4.4/1.7, a flip angle of 10°, a slice thickness of 1.2 mm and an acquisition time of 1 min. The images were obtained before and at 10, 70, 130, 190, 250 and 310 s after the injection of gadolinium DTPA (0.1 mmol/kg Gadovist; Bayer Schering Pharma, Berlin, Germany).

#### Appendix A.1.2. Ingenia, Philips Medical Systems

(1) Axial turbo spin-echo T2-weighted imaging with a TR/TE of 3919/80  ms, a flip angle of 90°, a field of view of 300 × 300 mm^2^, a matrix size of 484 × 300, 2 excitations, a slice thickness of 2 mm and an acquisition time of 3 min; (2) axial diffusion-weighted imaging with a single-shot spin-echo echoplanar image pulse sequence with a TR/TE of 12,043.5/102.3 ms, a flip angle of 90°, an FOV of 320 × 320 mm^2^, a matrix size of 184 × 184 and a slice thickness of 3 mm, using three b values (b = 0, 1000 s/mm2). The ADC map was calculated with an mono-exponential fit using a b value of 1000; (3) pre- and post-contrast T1-weighted high-resolution isotropic volume examination with a TR/TE of 4.0/1.8 ms, a field of view of 300 × 300 mm^2^, a matrix size 332 × 332, 1 excitation, a slice thickness of 1 mm, a flip angle of 12° and an acquisition time of 1 min 10 s. The images were obtained before and at 85, 155, 225, 295 and 365 s after the injection of 0.1 mmol/kg Gadovist, followed by a 20 mL saline flush.

### Appendix A.2. Clinicopathologic Information and Conventional MRI Analysis

Patient age, operations, adjuvant therapy after surgery, pathologic stage (T, N stage by AJCC 7th edition) and follow-up period after surgery were reviewed from medical records. Histologic type, tumor grade, hormone receptor status, Ki-67 index and the presence of lymphovascular invasion were collected from pathologic reports after surgery. When patients underwent neoadjuvant chemotherapy, the clinical stage was adopted, and the histologic information of the tumor was obtained from a biopsy report.

## Appendix C. Discussion

The margin of error can measure the amount of random sampling error in the survey data. Riley RD et al. [25] recommend that a margin of error should be smaller than or equal to 0.05 to build robust clinical prediction models. Especially when we have a binary outcome (recurrence/no-recurrence), a margin of error from an approximate 95% confidence interval is

$$1.96\sqrt{\frac{0.129\left(1-0.129\right)}{155}}=0.053,$$

where 0.129 is a sample recurrence rate, and 155 is our sample size.
